# Fast connectivity gradient approximation: maintaining spatially fine-grained connectivity gradients while reducing computational costs

**DOI:** 10.1038/s42003-024-06401-4

**Published:** 2024-06-06

**Authors:** Karl-Heinz Nenning, Ting Xu, Arielle Tambini, Alexandre R. Franco, Daniel S. Margulies, Stanley J. Colcombe, Michael P. Milham

**Affiliations:** 1 Nathan S. Kline Institute for Psychiatric Research, Orangeburg, NY USA; 2 Child Mind Institute, New York, NY USA; 3 New York University, New York, NY USA; 4 CNRS & Université de Paris, Paris, France

**Keywords:** Cognitive neuroscience, Network models

## Abstract

Brain connectome analysis suffers from the high dimensionality of connectivity data, often forcing a reduced representation of the brain at a lower spatial resolution or parcellation. This is particularly true for graph-based representations, which are increasingly used to characterize connectivity gradients, capturing patterns of systematic spatial variation in the functional connectivity structure. However, maintaining a high spatial resolution is crucial for enabling fine-grained topographical analysis and preserving subtle individual differences that might otherwise be lost. Here we introduce a computationally efficient approach to establish spatially fine-grained connectivity gradients. At its core, it leverages a set of landmarks to approximate the underlying connectivity structure at the full spatial resolution without requiring a full-scale vertex-by-vertex connectivity matrix. We show that this approach reduces computational time and memory usage while preserving informative individual features and demonstrate its application in improving brain-behavior predictions. Overall, its efficiency can remove computational barriers and enable the widespread application of connectivity gradients to capture spatial signatures of the connectome. Importantly, maintaining a spatially fine-grained resolution facilitates to characterize the spatial transitions inherent in the core concept of gradients of brain organization.

## Abstract (web summary)

This work presents a computationally efficient approach to establish spatially fine-grained connectivity gradients, reducing computational costs while preserving informative individual features and improving brain-behavior predictions.

## Introduction

Graph-based models have become mainstream tools for the visualization and characterization of relationship structures within complex systems in machine learning, computer vision, and bioinformatics. Unfortunately, their application is not without challenges—particularly when dealing with representations at higher resolutions, as their computational and storage demands can exceed the capacity of most computing resources. Data reduction is indispensable when the feasibility of graph-based models is impacted. Such reductions can be achieved through statistical techniques (e.g., principal component, factor analyses), data downsampling approaches (e.g., local averaging based on spatial or topographic structure), or both. Here, using graph-based models in neuroimaging as an example, we demonstrate the need for careful consideration of the order in which these two reduction strategies are applied, suggesting an optimized workflow when both are pursued.

In neuroimaging, graph-based representations are increasingly used to characterize topographic patterns of gradual transitions and boundaries between systems—i.e., connectivity gradients^1–3^. In contrast to typical functional connectivity analysis, which focuses on examining relationships between brain regions^4,5^, connectivity gradients capture patterns of systematic spatial variation in the functional connectivity structure^1,6^. They leverage an affinity matrix, which encodes the spatial similarities between region-wise connectivity profiles, to uncover the underlying low-dimensional principles of functional brain organization within the high-dimensional connectivity data. While functional connectivity focuses on the connections between specific brain regions, connectivity gradients provide a more global and continuous representation of functional brain organization^1,7^. But maintaining a full spatial representation of the connectome is challenging. The number of connections scales quadratically with the number of nodes, and a common spatial resolution of the brain comprising 60,000 cortical nodes (vertices) constitutes 1,799,970,000 pairwise connections (edges). This presents a substantial challenge to characterize the full-scale connectivity structure on consumer-grade hardware and without access to dedicated computational resources. A common practice to overcome this challenge is to reduce the spatial resolution of the data, typically by averaging across vertices within spatial regions of interest (ROIs) defined using a parcellation template^8^. However, the loss of individual-specific detail in such parcellation approaches may result in the loss of meaningful information; there is also little agreement on the appropriate choice of parcellation templates in connectome studies^9,10^. Moreover, the capability to maintain a fine-grained spatial resolution allows researchers to fully characterize the individualized spatial layout of functional regions, an important and often overlooked aspect of modeling brain connectivity^11–13^, which could improve brain-behavior associations. The emphasis on spatial specificity aligns with the core principle of connectivity gradients—capturing axes of systematic connectivity change which delineate gradual transitions across brain areas^7^. Discrete parcellation schemes might obscure the topography of such smooth transitions and unclear border regions, failing to fully capture the continuous and gradual nature of connectivity gradients^1^.

Here, to facilitate connectivity gradients at a fine-grained spatial resolution, we propose Fast Connectivity Gradient Approximation (FCGA). Instead of using a full-scale vertex-by-vertex connectivity matrix, at its core, FCGA leverages a set of landmarks to approximate the underlying connectivity structure at full spatial resolution, with an efficiency that makes it possible to run on common computer hardware. These landmarks are flexible, and can, for example, be based on individual vertices or predefined ROIs.

## Results and discussion

**Fig. 1:**
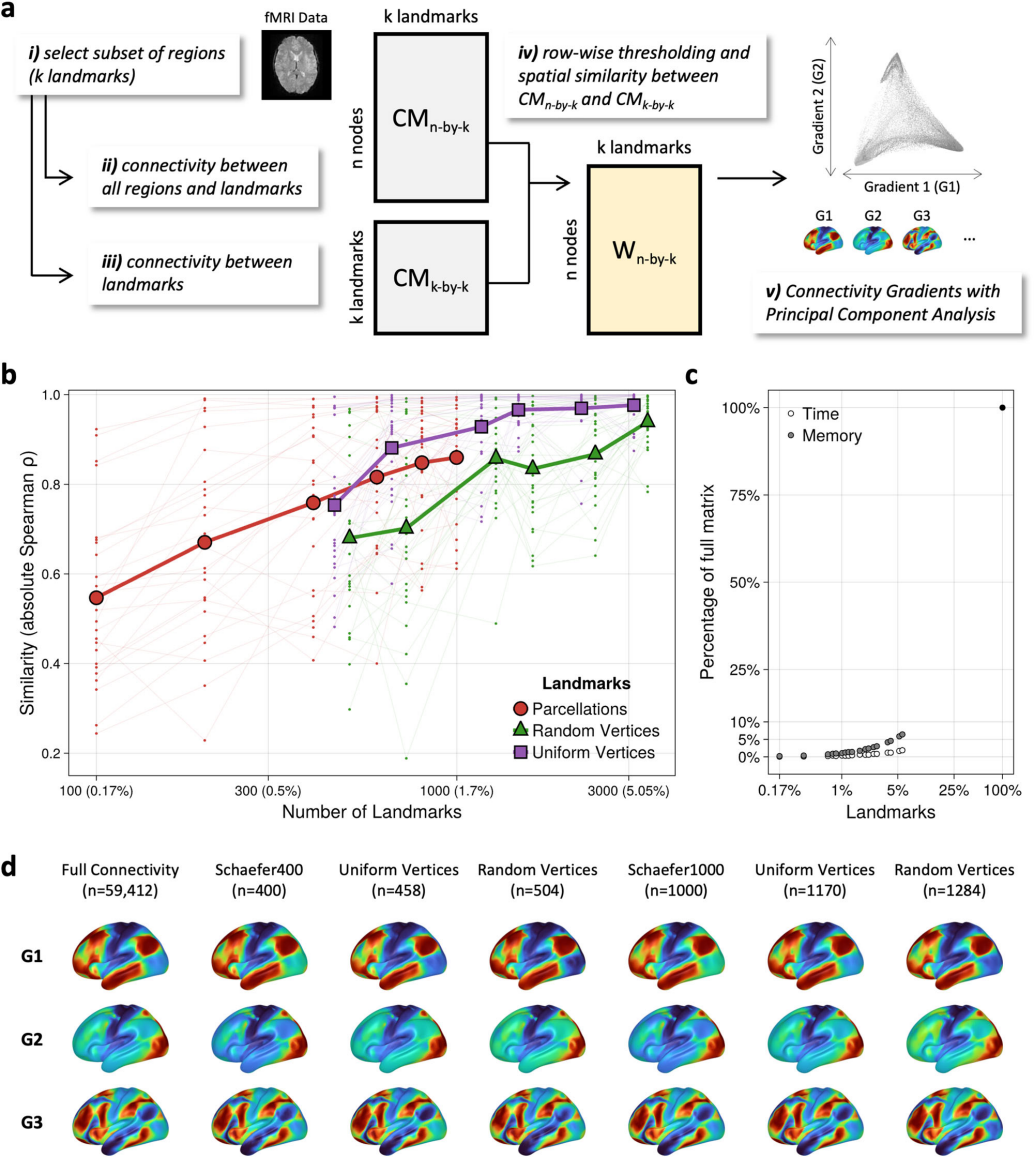
Gradient approximation facilitates spatially fine-grained connectivity gradients while reducing computational costs. **a** A schematic workflow of the proposed Fast Connectivity Gradient Approximation (FCGA) approach. **b** FCGA provides high fidelity to the full connectivity structure with only a fraction of landmarks. Thin line plots denote the spatial similarity of the first 25 gradients, and thick line plots denote the respective average. **c** FCGA facilitates a high spatial similarity with reduced computational time and memory requirements. **d** The core topography is similar across a different number of landmarks and sampling choices.

A schematic workflow of our proposed approach is displayed in Fig. 1a. After (i) establishing a set of k landmarks, the (ii) functional connectivity between all n vertices (or voxels) and the k landmarks is calculated, resulting in a n-by-k connectivity matrix (CM_n-by-k_) between all pairs of vertices and landmarks. Following current practices when calculating connectivity gradients^1,14^, we quantify the spatial similarity of vertex-wise connectivity profiles. To do so, (iii) a k-by-k (landmark-by-landmark) connectivity matrix (CM_k-by-k_) that characterizes the functional connectivity between all pairs of landmarks is established. Then, row-wise thresholding, retaining the 10% strongest positive connections, is applied to both CM_n-by-k_ and CM_k-by-k_. Subsequently, (iv) the row-wise cosine similarity between all pairs of vertices in CM_n-by-k_ and CM_k-by-k_ is calculated. This results in an n-by-k affinity matrix (W_n-by-k_) that characterizes the spatial similarities between the voxel-wise connectivity profiles with the landmark regions. Finally, (v) we use Principal Component Analysis (PCA) for dimensionality reduction of W_n-by-k_, establishing the approximated connectivity gradients.

We evaluated the validity of FCGA on the group and individual level, using high-resolution fMRI data (59,412 cortical vertices) from the Human Connectome Project (HCP)^15^ and the Nathan Kline Institute-Rockland Sample (NKI-RS)^16^. We parametrically varied the amount of downsampling (number of landmarks) across sampling choices (vertex- and ROI-level).

At the group level, we used Spearman rank correlation to compare the spatial similarity between the approximated connectivity gradients (G_FCGA_) and the connectivity gradients based on the full 59,412-by-59,412 connectivity matrix (G_full_fc_). The spatial similarity between G_FCGA_ and G_full_fc_ increased with the number of landmarks used to calculate the approximated gradients (Fig. 1b). Remarkably, using 1000 ROIs as landmarks (~1.7% of the full connectivity matrix), the average spatial similarity across 25 connectivity gradients reached *⍴* = 0.86. Using 3000 (~5%) uniformly distributed vertices, a spatial similarity of *⍴* = 0.98 was achieved with <10% of the computational time and memory usage as compared to the calculation of G_full_fc_ (Fig. 1c). This was replicated on the individual level, where across 100 individuals, G_FCGA_ with landmarks based on ~5% uniformly distributed vertices yielded an average spatial similarity of *⍴* = 0.9 (Supplementary Fig. S1). The spatial topography of the top connectivity gradients, shown in Fig. 1d, further illustrates the high spatial similarity between G_FCGA_ and G_full_fc_ across a number of sampling choices. Vertex-wise similarities between the gradient profiles of G_FCGA_ and G_full_fc_ further emphasized the accuracy of the gradient approximation (Supplementary Fig. S2).

**Fig. 2:**
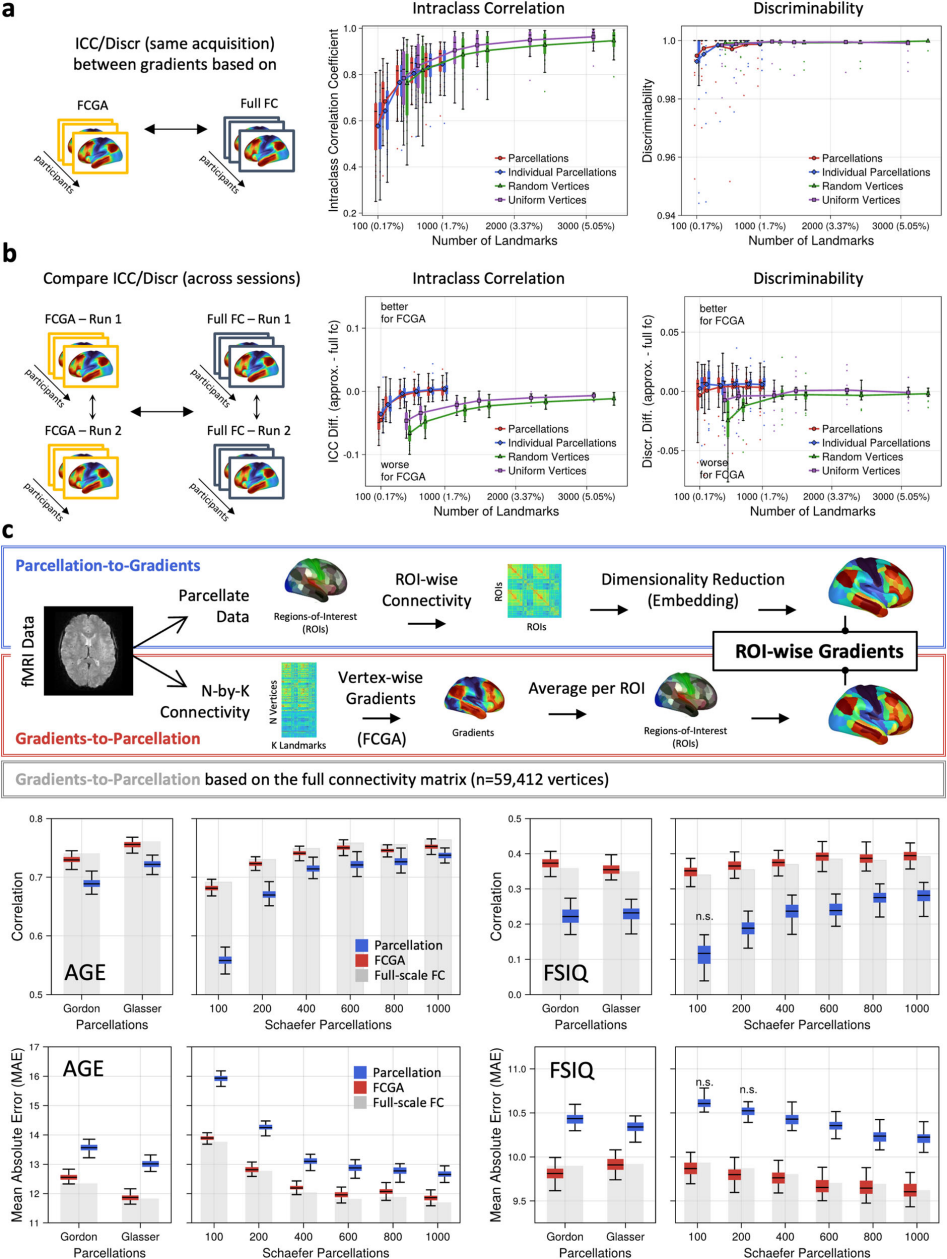
Gradient approximation preserves informative individual features and improves brain-behavior predictions. Intraclass correlation and discriminability analysis confirms the reliability, repeatability, and the preservation of individual features (**a**) within and (**b**) across sessions. Boxplots show intraclass correlation and discriminability for the first 25 gradients, and colored boxes indicate interquartile range (iqr) with whiskers spanning 1.5*iqr. Markers and line plots denote respective means. **c** Parcel-averaging of spatially fine-grained gradients outperforms gradient calculation on a parcel level for both age and FSIQ prediction. Fast connectivity gradient approximation (FCGA) with landmarks based on the group-average Schaefer parcellation (*n* = 1000) was used to establish vertex-level gradients before parcel averaging. The first five gradients were used as features. Boxplots show 100 runs of tenfold cross-validation, and colored boxes indicate interquartile range (iqr) with whiskers spanning 1.5*iqr. Predictions that are not significantly better than prediction with randomly shuffled labels are denoted as n.s.

At the individual level, reliability and discriminability analysis confirmed the repeatability and the preservation of individual features with G_FCGA_. To quantify the agreement between G_FCGA_ and G_full_fc_, we calculated the intraclass correlation coefficient (ICC) and discriminability for the same acquisition. We observed an average vertex-wise ICC of >0.9 from 1000 (~1.7%) landmarks onwards, and discriminability was already nearly perfect with 300 (<1%) landmarks (Fig. 2a). Next, we compared the ICC and the discriminability of G_FCGA_ and G_full_fc_ across sessions (Fig. 2b). We observed that landmarks based on single vertices (uniformly or randomly distributed) yielded slightly lower ICC and discriminability for G_FCGA_ than for G_full_fc_. However, landmarks based on predefined parcels on the group or individual level yielded a slightly better discriminability and a similar ICC for G_FCGA_ when compared to G_full_fc_ (Fig. 2b).

Finally, we evaluated the practical implications of our FCGA approach on brain-wide association studies by predicting age and full-scale intelligence (FSIQ) across the lifespan using the first five gradients. Specifically, we compared the predictive performance of coarse-grained gradients calculated from parcellated time-series to FCGA constructed parcel-wise summarized fine-grained gradients (Fig. 2c). Importantly, we observed that fine-grained gradients (i.e., G_FCGA_) subjected to parcel-averaging outperformed coarse-grained gradients for predicting both age and FSIQ across all tested parcellations (p_Bonferroni _< 0.05 in 32/32 comparisons) (Fig. 2c). Notably, for FSIQ we observed that gradients calculated on a low number of parcels did not perform better than chance. The improved prediction for parcel averaging of spatially fine-grained gradients was replicated in the HCP sample across age-adjusted composite cognition scores and an estimate for fluid intelligence (Supplementary Fig. S3). This indicates that constructing connectivity gradients from parcellated data might lose meaningful information in the process that is otherwise preserved in the fine-grained gradient topography.

The proposed approximation framework is not limited to the landmarks we used in the current study and is flexible to incorporate study-specific landmarks if needed. For example, landmarks based on individualized parcellations might yield higher discriminability across sessions, indicating potential benefits of optimized landmarks for capturing individual differences. While identifying optimal regional homologies across development or aging requires further research, we demonstrated that the use of uniformly or randomly distributed vertices as landmarks already offers a highly accurate approximation on the group and individual levels.

It is also important to note that a vertex-wise representation is not necessarily superior to parcellation-based representations when the quality of the data is suboptimal^17,18^. Region-wise averaging helps to increase the signal-to-noise ratio, and spatially fine-grained data can entail computational challenges for further analyses. Nonetheless, our findings indicate that summarizing the gradient coefficients of a vertex-level representation is more informative than calculating the gradients directly on parcellated (downsampled) data. While future research is necessary to evaluate the potential applications for connectivity gradients^14,19^, an intermediate vertex-level representation might benefit further parcellation-based gradient analysis.

Taken together, our results demonstrate the feasibility of establishing spatially fine-grained connectivity gradients while mitigating the computational burden of vertex-level functional connectivity data by reducing computational time and memory usage. The ability to fully appreciate the spatial layout of functional regions in gradient representations at full resolution could improve associations between functional imaging data and cognitive traits^11^, and inform structure-function relationships^20^. Importantly, the advantage of preserving informative individual features in gradients of spatially fine-grained connectivity data was emphasized by the improved prediction of age and intelligence over gradients based on coarse-grained connectivity data. Overall, FCGA strengthens the core concept of connectivity gradients by maintaining the full spatial resolution to study spatial transitions of brain organization. Finally, its efficiency also removes computational barriers and can enable the widespread application of connectivity gradients to capture signatures of the connectome.

## Methods

### Data

To evaluate the validity of FCGA on the group and individual levels, we used resting-state fMRI data from the HCP^15^. The HCP data was acquired at Washington University at St. Louis on a customized Siemens 3T Connectome Skyra scanner. Institutional Review Board approval was obtained at the Washington University in St Louis, and written informed consent was obtained for all study participants. All ethical regulations relevant to human research participants were followed. Resting-state fMRI was acquired with a multiband factor of 8, 2 mm isotropic resolution, and a repetition time of 0.72 s for a duration of 14.4 min, resulting in 1200 volumes per acquisition. Participants were asked to relax, keep eyes open, and fixated on a crosshair, and not to fall asleep. Four resting-state runs were collected in different sessions across two days (REST1 and REST2), where each session comprised two runs with different phase encoding directions (LR and RL). We used the minimally preprocessed fMRI data provided by the HCP^21^. Briefly, the resting-state data has been motion corrected, minimally spatially smoothed (2 mm), high-pass filtered (2000s cutoff), denoised for motion-related confounds and artifacts using independent component analysis^22^, and spatially aligned to the 2 mm standard CIFTI grayordinates space^21,23^.

At the group level, we used the dense group-average functional connectome provided as part of the HCP S1200 data release. In brief, this connectivity matrix was constructed from the minimal preprocessed data across 1003 individuals that each had ~1 h (4 × 14.4 min) of resting-state fMRI acquisitions. The dense connectivity matrix of size 91,282 × 91,282 grayordinates (59,412 cortical vertices and 31,870 subcortical voxels) was calculated based on components of an incremental group-PCA. For the group-average connectome of the HCP, we averaged the precomputed functional connectivity measures for each landmark instead of recalculating landmark-based connectivity. At individual level, we used the 100 unrelated subjects cohort from the HCP (54F/46M, age = 29 ± 3.7 years) and two repeated resting-state fMRI runs (REST1_LR and REST2_LR) for each individual. We used the minimally preprocessed data as provided by the HCP. For both the group and individual levels, we focused our analysis on the 59,412 cortical vertices.

We used a cross-sectional lifespan sample with 313 healthy participants (214 female, age: 6–85 years, 42.2 ± 22.4 years) to evaluate the practical implications of our approach on brain-wide association studies. Participants were selected from the NKI-RS^16^, who have no diagnosis of any mental or neurological disorders and passed quality control of a head motion criteria (mean framewise displacement < 0.25 mm). The NKI-RS data was acquired at the Nathan Kline Institute on a Siemens TrioTim 3 Tesla scanner. Institutional review board approval was obtained at the Nathan Kline Institute, and written informed consent was obtained for all study participants. All ethical regulations relevant to human research participants were followed. Resting-state fMRI was acquired with a multiband factor of 4, 3 mm isotropic resolution, and a repetition time of 0.645 s for a duration of 9.7 min, which resulted in 900 volumes per run. Preprocessing was performed with the Connectome Computational System^24^ and included discarding the first five time points, compressing temporal spikes, slice timing correction, motion correction, 4D global mean intensity normalization, nuisance regression (Friston’s 24 model, cerebrospinal fluid and white matter), linear and quadratic detrending, band-pass filtering (0.01–0.1 Hz), as well as global signal regression. The preprocessed data were then projected on the 32k fsLR surface template with 32,492 vertices per hemisphere.

### Connectivity landmarks

We used a varying number of landmarks across different sampling strategies. We utilized randomly and uniformly distributed vertices across the cortex, where the selected vertices were consistent across individuals. On the ROI-level, connectivity landmarks were defined by the average time series based on group parcellations^25–27^ and individualized parcellations for the HCP sample^28^.

### Comparing gradients

For the group-level analysis and comparisons, no alignment or reordering of the gradients (PCA components) was performed. For the analysis on the individual level, we used one set of reference gradients based on the HCP dense connectome for both the approximated and full-scale gradients. For each individual, sampling, and gradient construction approaches, the gradients were aligned to this reference with orthogonal Procrustes alignment^29^. Orthogonal Procrustes finds the optimal linear transformation so that two sets of gradients have a matched component order and coefficient signs.

### Intraclass correlation and discriminability

We compared the reliability and discriminability of the approximated gradients (G_FCGA_) and the full-scale gradients (G_full_fc_) within and across sessions, using the two repeated resting-state acquisitions from the 100 unrelated individual samples of the HCP. Within-session analysis treated G_FCGA_ and G_full_fc_ of the same acquisition as repeated measures. Across-session analysis evaluated differences in the reliability and reproducibility between G_FCGA_ and G_full_fc_. Discriminability^30^ was used to measure the similarity of connectivity gradients. It is a nonparametric multivariate statistic that quantifies the degree to which repeated measurements (e.g., gradients of different approaches or sessions) are relatively similar to each other. At the vertex level, we quantified the ICC, a univariate measure of the degree of absolute agreement^31,32^.

### Prediction of individual-specific measures

We used the NKI-RS lifespan sample to evaluate implications of our proposed approach for the prediction of individual-specific measures such as age and FSIQ, which were only weakly correlated (*r* = 0.13). As described above, connectivity gradients are often calculated on a reduced data representation on a parcel level. In this study, we compared the predictive performance of connectivity gradients calculated on parcellated time series (parcellation-to-gradients) to a parcel-wise averaging of fine-scale gradients constructed with FCGA (gradients-to-parcellation). Connectivity gradients of the parcellated data are established as in prior work^1^, using all parcels as landmarks instead of a subset. We evaluated distinct, commonly used parcellations^25–27^, and focused on the first five gradients as features to avoid excessively increasing the feature space. For prediction, we used ridge regression with a L2 regularization as implemented with Glmnet^33^, and a nested tenfold cross-validation scheme for hyperparameter (lambda) selection. The predictions of each fold were aggregated, and performance was measured with mean absolute error and correlation to the true age and FSIQ values. We repeated the tenfold cross-validation runs 100 times, with random splits for each fold. Additionally, we tested the prediction results against a baseline of 100 prediction runs with randomly shuffled labels to evaluate if the predictive performance is greater than chance.

### Statistics and reproducibility

Spatial similarity between the approximated connectivity gradients (G_FCGA_) and the connectivity gradients based on the full connectivity matrix (G_full_fc_) was quantified using Spearman correlation at both group- and individual levels. A tenfold cross-validation scheme was used for the predictive modeling, utilizing a nested cross-validation within the training set for hyperparameter selection. The tenfold cross-validation was repeated 100 times with random splits. Predictive modeling was performed on the NKI-RS lifespan sample and replicated with the HCP sample.

### Reporting summary

Further information on research design is available in the Nature Portfolio Reporting Summary linked to this article.

### Supplementary information

Supplementary Information

Description of Additional Supplementary Materials

Supplementary Data 1

Reporting Summary

## Data Availability

The dataset used in this work are publicly available at the Human Connectome Project (https://www.humanconnectome.org) and the enhanced Nathan Kline Institute-Rockland Sample data repository (https://fcon_1000.projects.nitrc.org/indi/enhanced). The source data underlying all figures in the manuscript can be found in the Supplementary Data.

## Code availability

The preprocessing code for the NKI data is available at https://github.com/zuoxinian/CCS, the code for discriminability is available at https://github.com/neurodata/discriminability, and for intraclass coefficient at https://github.com/TingsterX/Reliability_Explorer. Glmnet is available at https://glmnet.stanford.edu/index.html. The Fast Connectivity Gradient Approximation code is available at https://github.com/khne/FastConnectivityGradientApproximation.
