# Supplementary material for: Fast connectivity gradient approximation: maintaining spatially fine-grained connectivity gradients while reducing computational costs
Source: Commun Biol. 2024 Jun 6;7:697. doi: 10.1038/s42003-024-06401-4 (PMC11156950; doi:10.1038/s42003-024-06401-4)
Supplement: Supplementary file 1 — Supplementary Information [file 42003_2024_6401_MOESM1_ESM.pdf]

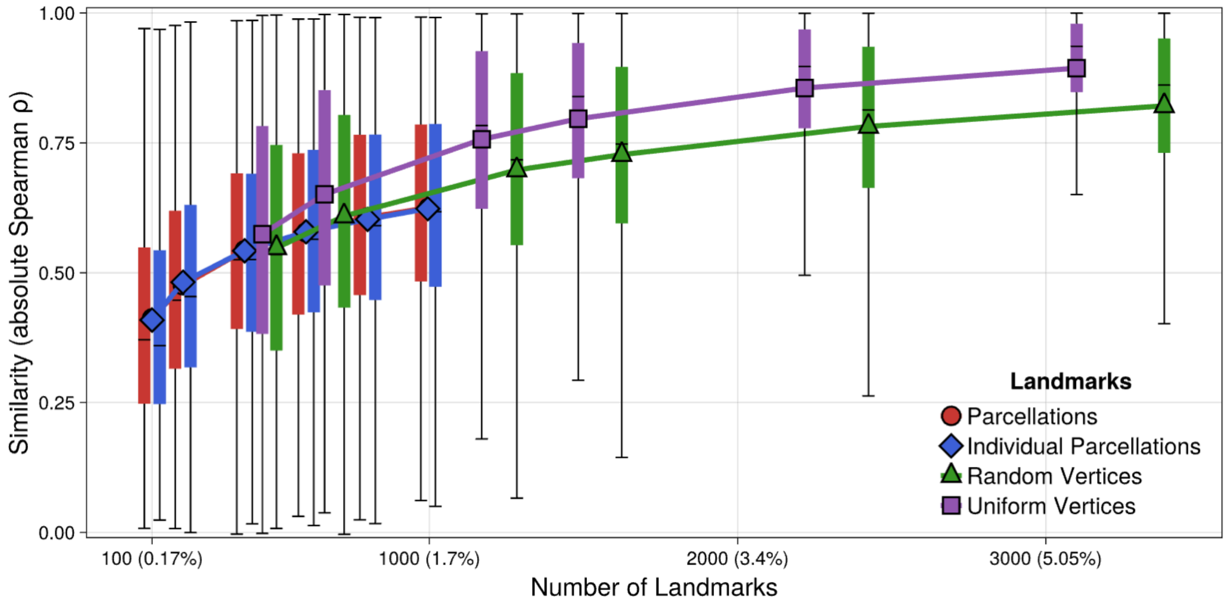

Supplementary Figure S1. The spatial similarity between the proposed Fast Connectivity Gradient Approximation (FCGA) approach and gradients based on the full connectivity structure across the HCP 100 unrelated individuals subset. A high similarity is achieved with only a fraction of landmarks. Boxplots show spatial similarities for 25 gradients across 100 individuals, and colored boxes indicate interquartile range (iqr) with whiskers spanning 1.5*iqr. Markers and lines denote respective means.

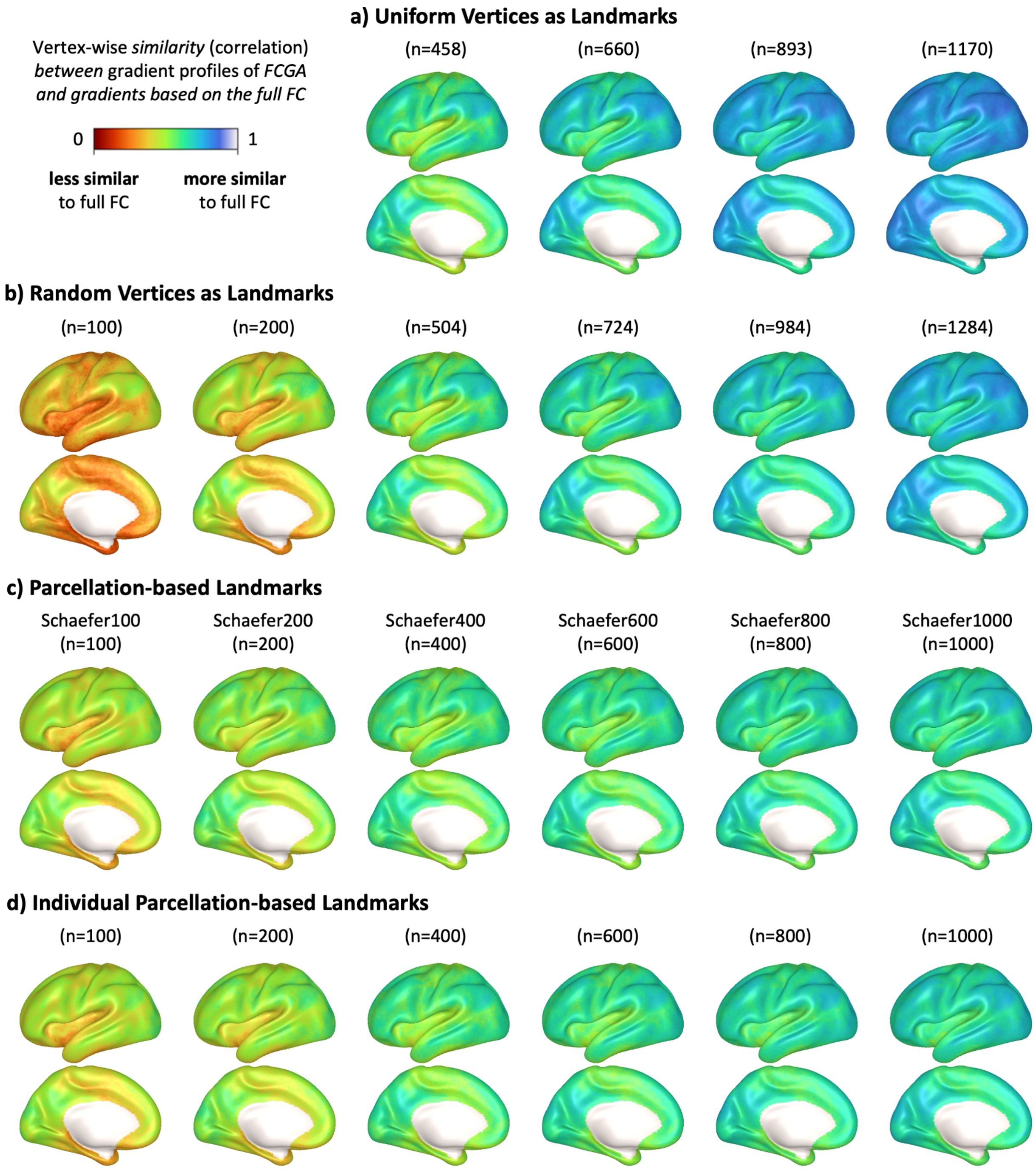

Supplementary Figure S2. Vertex-wise similarities between approximated gradients and gradients based on the full connectivity matrix. Similarity was calculated as the correlation between vertex-wise gradient profiles over 25 gradients, where each gradient was z-scored to account for different gradient scales.

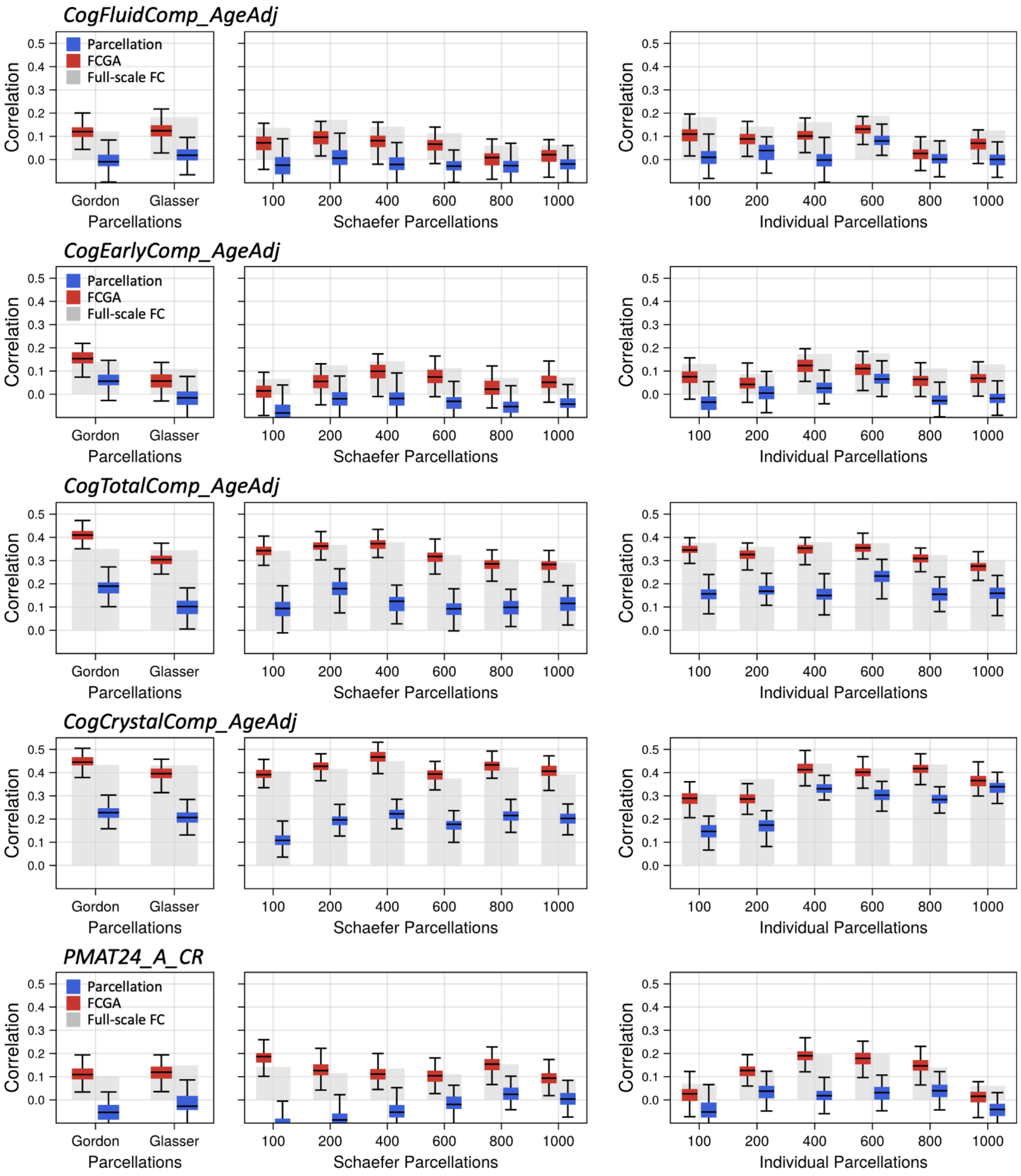

Supplementary Figure S3. Comparing the predictive performance of parcel-averaged spatially fine-grained gradients to gradients calculated directly on parcellated data in the HCP 100 unrelated subjects subcohort (REST1_LR and REST2_LR). Fast connectivity gradient approximation (FCGA) with landmarks based on the group-average Schaefer parcellation (n=1000) was used to establish vertex-level gradients before parcel-averaging. The first five gradients were used as features. Boxplots show 100 runs of 10-fold cross-validation, and colored boxes indicate interquartile range (iqr) with whiskers spanning 1.5*iqr.
