# Supplementary material for: Fast connectivity gradient approximation: maintaining spatially fine-grained connectivity gradients while reducing computational costs
Source: Commun Biol. 2024 Jun 6;7:697. doi: 10.1038/s42003-024-06401-4 (PMC11156950; doi:10.1038/s42003-024-06401-4)
Supplement: Supplementary file 2 — Description of Additional Supplementary Materials [file 42003_2024_6401_MOESM2_ESM.docx]

**Description of Additional Supplementary Files**

**File name:** Supplementary Data 1

**Description:** The source data underlying the graphs in the paper
