# Supplementary material for: Fast connectivity gradient approximation: maintaining spatially fine-grained connectivity gradients while reducing computational costs
Source: Commun Biol. 2024 Jun 6;7:697. doi: 10.1038/s42003-024-06401-4 (PMC11156950; doi:10.1038/s42003-024-06401-4)
Supplement: Supplementary file 4 — Reporting Summary [file 42003_2024_6401_MOESM4_ESM.pdf]

nature portfolio

Corresponding author(s): Karl-Heinz Nenning

Last updated by author(s): May 14, 2024

Reporting Summary

Nature Portfolio wishes to improve the reproducibility of the work that we publish. This form provides structure for consistency and transparency in reporting. For further information on Nature Portfolio policies, see our Editorial Policies and the Editorial Policy Checklist.

Statistics

For all statistical analyses, confirm that the following items are present in the figure legend, table legend, main text, or Methods section.

n/a	Confirmed	
☐	☒	The exact sample size (*n*) for each experimental group/condition, given as a discrete number and unit of measurement
☐	☒	A statement on whether measurements were taken from distinct samples or whether the same sample was measured repeatedly
☐	☒	The statistical test(s) used AND whether they are one- or two-sided<br>*Only common tests should be described solely by name; describe more complex techniques in the Methods section.*
☐	☒	A description of all covariates tested
☒	☐	A description of any assumptions or corrections, such as tests of normality and adjustment for multiple comparisons
☒	☐	A full description of the statistical parameters including central tendency (e.g. means) or other basic estimates (e.g. regression coefficient) AND variation (e.g. standard deviation) or associated estimates of uncertainty (e.g. confidence intervals)
☒	☐	For null hypothesis testing, the test statistic (e.g. *F*, *t*, *r*) with confidence intervals, effect sizes, degrees of freedom and *P* value noted<br>*Give P values as exact values whenever suitable.*
☒	☐	For Bayesian analysis, information on the choice of priors and Markov chain Monte Carlo settings
☒	☐	For hierarchical and complex designs, identification of the appropriate level for tests and full reporting of outcomes
☒	☐	Estimates of effect sizes (e.g. Cohen's *d*, Pearson's *r*), indicating how they were calculated

Our web collection on statistics for biologists contains articles on many of the points above.

Software and code

Policy information about availability of computer code

Data collection	No data collection was performed. We reused data from the publicly available Human Connectome Project (HCP; https://www.humanconnectome.org) and the Nathan Kline Institute-Rockland Sample data repository (NKI-RS; http://fcon_1000.projects.nitrc.org/indi/enhanced/).
Data analysis	Data analysis was performed with Julia v1.7.3 and Python v3.10. The preprocessing code for the NKI data is available at https://github.com/zuoxinian/CCS, the code for discriminability is available at https://github.com/neurodata/discriminability, and for intraclass coefficient at https://github.com/TingsterX/Reliability_Explorer. Glmnet is available at https://glmnet.stanford.edu/index.html. The Fast Connectivity Gradient Approximation code is available at https://github.com/khne/FastConnectivityGradientApproximation.

For manuscripts utilizing custom algorithms or software that are central to the research but not yet described in published literature, software must be made available to editors and reviewers. We strongly encourage code deposition in a community repository (e.g. GitHub). See the Nature Portfolio guidelines for submitting code & software for further information.

Data

Policy information about availability of data

All manuscripts must include a data availability statement. This statement should provide the following information, where applicable:

- Accession codes, unique identifiers, or web links for publicly available datasets
- A description of any restrictions on data availability
- For clinical datasets or third party data, please ensure that the statement adheres to our policy

Data used in this work are publicly available at the Human Connectome Project (https://www.humanconnectome.org) and the Nathan Kline Institute-Rockland Sample data repository (http://fcon_1000.projects.nitrc.org/indi/enhanced/).

Research involving human participants, their data, or biological material

Policy information about studies with human participants or human data. See also policy information about sex, gender (identity/presentation), and sexual orientation and race, ethnicity and racism.

Reporting on sex and gender	The HCP sample consists of 54 female and 46 male participants, and the NKI-RS cohort of 214 female and 99 male participants. Sex was reported in the methods section.
Reporting on race, ethnicity, or other socially relevant groupings	*Please specify the socially constructed or socially relevant categorization variable(s) used in your manuscript and explain why they were used. Please note that such variables should not be used as proxies for other socially constructed/relevant variables (for example, race or ethnicity should not be used as a proxy for socioeconomic status).* *Provide clear definitions of the relevant terms used, how they were provided (by the participants/respondents, the researchers, or third parties), and the method(s) used to classify people into the different categories (e.g. self-report, census or administrative data, social media data, etc.)* *Please provide details about how you controlled for confounding variables in your analyses.*
Population characteristics	The HCP cohort consists of 100 unrelated young adults (age: mean 29 ± 3.7 years). The NKI-RS cohort consists of a 313 healthy participants from a community lifespan sample (age: 6-85 years, mean 42.2 ± 22.4 years)
Recruitment	The Human Connectome Project aimed to recruit directly-related participants of 300 families. The Enhanced Nathan Kline Institute - Rockland Sample aimed to recruit a representative community sample from Rockland County, whose ethnic and economic demographics resemble those of the United States, increasing the generalizability of the NKI-RS to the broader U.S. population.
Ethics oversight	For the HCP data, IRB approval was granted to the WU-Minn HCP consortium. For the NKI-RS project, IRB approval was obtained at the Nathan Kline Institute and at Montclair State University.

Note that full information on the approval of the study protocol must also be provided in the manuscript.

Field-specific reporting

Please select the one below that is the best fit for your research. If you are not sure, read the appropriate sections before making your selection.

☒ Life sciences ☐ Behavioural & social sciences ☐ Ecological, evolutionary & environmental sciences

For a reference copy of the document with all sections, see nature.com/documents/nr-reporting-summary-flat.pdf

Life sciences study design

All studies must disclose on these points even when the disclosure is negative.

Sample size	Sample size was determined by the size of the used datasets.
Data exclusions	No data was excluded from the HCP 100 unrelated individuals sample. From the NKI-RS cohort, we selected participants that have no diagnosis of any mental or neurological disorders, and passed quality control of a head motion criteria (mean framewise displacement < 0.25mm).
Replication	Not applicable
Randomization	Not applicable
Blinding	Not applicable

Reporting for specific materials, systems and methods

We require information from authors about some types of materials, experimental systems and methods used in many studies. Here, indicate whether each material, system or method listed is relevant to your study. If you are not sure if a list item applies to your research, read the appropriate section before selecting a response.

Materials & experimental systems

n/a	Involved in the study
☒	☐ Antibodies
☒	☐ Eukaryotic cell lines
☒	☐ Palaeontology and archaeology
☒	☐ Animals and other organisms
☒	☐ Clinical data
☒	☐ Dual use research of concern
☒	☐ Plants

Methods

n/a	Involved in the study
☒	☐ ChIP-seq
☒	☐ Flow cytometry
☐	☒ MRI-based neuroimaging

Plants

Seed stocks

Report on the source of all seed stocks or other plant material used. If applicable, state the seed stock centre and catalogue number. If plant specimens were collected from the field, describe the collection location, date and sampling procedures.

Novel plant genotypes

Describe the methods by which all novel plant genotypes were produced. This includes those generated by transgenic approaches, gene editing, chemical/radiation-based mutagenesis and hybridization. For transgenic lines, describe the transformation method, the number of independent lines analyzed and the generation upon which experiments were performed. For gene-edited lines, describe the editor used, the endogenous sequence targeted for editing, the targeting guide RNA sequence (if applicable) and how the editor was applied.

Authentication

Describe any authentication procedures for each seed stock used or novel genotype generated. Describe any experiments used to assess the effect of a mutation and, where applicable, how potential secondary effects (e.g. second site T-DNA insertions, mosiacism, off-target gene editing) were examined.

Magnetic resonance imaging

Experimental design

Design type

resting-state fMRI

Design specifications

The HCP data was acquired at Washington University at St. Louis on a customized Siemens 3T Connectome Skyra scanner. Resting-state fMRI was acquired with a multiband factor of 8, 2mm isotropic resolution, and a repetition time of 0.72 seconds for a duration of 14.4 minutes, resulting in 1200 volumes per acquisition. Participants were asked to relax, keep eyes open and fixated on a crosshair, and not to fall asleep. The NKI-RS data was acquired at the Nathan Kline Institute on a Siemens TrioTim 3 Tesla scanner. Resting-state fMRI was acquired with a multiband factor of 4, 3mm isotropic resolution, and a repetition time of 0.645 seconds for a duration of 9.7 minutes, which resulted in 900 volumes per run.

Behavioral performance measures

Not applicable

Acquisition

Imaging type(s)

structural, functional

Field strength

3 Tesla

Sequence & imaging parameters

HCP Data:
T1: 3D MPRAGE, 2400ms TR, 2.14ms TE, 1000ms TI, flip angle 8deg, 224x224mm FOV, 0.7mm isotropic voxel size
Resting state: Gradient-echo EPI, 720ms TR, 33.1ms TE, Flip Angle 52deg, 208x180mm FOV, 104x90 Matrix, 2.0mm slice thickness, 72 slices, 2.0mm isotropic voxels, multiband factor 8, 14:33(min:sec) duration

NKI-RS Data:
T1: 3D MPRAGE, 1900ms TR, 2.52ms TE, 900ms TI, flip angle 9deg, 250x250mm FOV, 1mm isotropic voxel size
Resting state: Gradient-echo EPI, 645ms TR, 30ms TE, Flip Angle 60deg, 240x240mm FOV, 74x74 Matrix, 3.0mm slice thickness, 40 slices, 3.0mm isotropic voxels, multiband factor 4, 9:46(min:sec) duration

Area of acquisition

whole brain

Diffusion MRI ☐ Used ☒ Not used

Preprocessing

Preprocessing software	The HCP data was preprocessed by the HCP consortium using the HCP preprocessing pipeline. The NKI-RS data was preprocessed using the Connectome Computation System.
Normalization	For the HCP data, non-linear spatial alignment to the 2 mm standard CIFTI grayordinates space was performed. The NKI-RS data was aligned to the FreeSurfer fsaverage space.
Normalization template	Conte69 fsaverage_LR surface template
Noise and artifact removal	Data was motion corrected. Artifacts in the HCP data were removed with ICA+FIX , and for the NKI-RS data nuisance regression with 6 head motion parameters, 6 head motion parameters one time point before, their squared values, and average signals for cerebrospinal fluid and white matter.
Volume censoring	no censoring was performed

Statistical modeling & inference

Model type and settings	functional connectivity gradients
Effect(s) tested	Not applicable

Specify type of analysis: ☒ Whole brain ☐ ROI-based ☐ Both

Statistic type for inference<br>(See Eklund et al. 2016)	Not applicable
Correction	Not applicable

Models & analysis

n/a	Involved in the study
☐	☒ Functional and/or effective connectivity
☒	☐ Graph analysis
☐	☒ Multivariate modeling or predictive analysis

Functional and/or effective connectivity	Pearson correlation
Multivariate modeling and predictive analysis	Coefficients of the connectivity gradients were averaged for regions of interest to reduce the dimensionality. In a nested nested 10-fold cross-validation scheme, a ridge regression model (glmnet) with a L2 regularization was used to predict age and fsiq. The 10-fold cross-validation run was repeated 100 times with random splits for each fold. To evaluate if the predictive performance is greater than chance, the prediction results were compared against a baseline of 100 prediction runs with randomly shuffled labels.
